# High-throughput amplicon sequencing datasets of microbial community in soils irrigated by quicklime and fly ash-treated acid mine drainage water

**DOI:** 10.1016/j.dib.2023.109849

**Published:** 2023-11-26

**Authors:** Henry Joseph Oduor Ogola, Rabelani Munyai, Ramganesh Selvarajan

**Affiliations:** a Department of Environmental Sciences, University of South Africa, Florida Science Campus; Roodepoort, 1709, South Africa; b Department of Agriculture and Animal Health, University of South Africa, Florida Science Campus; Roodepoort, 1709, South Africa; c Laboratory of Extraterrestrial Ocean Systems (LEOS), Institute of Deep-Sea Science and Engineering, Chinese Academy of Sciences, Sanya, PR China

**Keywords:** Soil microbiome, MOTHUR, Illumina, Treated acid mine drainage water, Soil health, Irrigated agriculture

## Abstract

In water-stressed regions, the use of treated acid mine drainage (AMD) water for irrigated agriculture has been suggested as an alternative to address the shortage of fresh water sources. However, the short and long-term impact of using such (un)treated AMD water on soil health, particularly the microbiome structure and functional capacity, is not known. We present high-throughput amplicon sequence (HTS) datasets of purified microbial metacommunity DNA of soils under Irish potato production irrigated by quicklime and fly ash treated AMD water. The irrigation treatments included quicklime treated AMD water (A1Q and A2Q; n = 16), and quicklime and fly ash-treated AMD water (AFQ; n = 5), untreated AMD water (uAMD; n = 7) and control group using tap water (n = 5). The V1-V3 hypervariable region of the 16S rRNA gene from each sample were sequenced on an Illumina MiSeq to generate these HTS datasets. The raw sequences underwent quality-checking, demultiplexing into FASTQ files, and processing using MOTHUR pipeline (v1.40.0). Th quality reads classified into taxonomic ids (phylum, class, order, family, and genus) using the Naïve Bayesian classifier algorithm against the SILVA database (v132) and were assigned to operational taxonomic units (OTUs) based on the pairwise distance matrix (Euclidean distance matrix). The applicability of the HTS datasets was confirmed by microbial taxa at the phylum level. All HTS datasets are available through the BioSample Submission Portal under the BioProject ID PRJNA974836 (https://www.ncbi.nlm.nih.gov/bioproject/974836).

Specifications Table

**Table:** 

Subject	Microbial Ecology
Specific subject area	Microbiology: Microbiome, High-throughput amplicon metagenome sequence datasets of the 16S rRNA gene (V1-V3 hypervariable segment) bulk soils of agricultural plots producing Irish potato under irrigation with quicklime-treated AMD water (A1Q and A2Q), quicklime and fly ash- treated AMD water (AFQ), untreated AMD water (uAMD) and tap water (Control)
Type of data	Figures and Tables
How the data were acquired	Illumina MiSeq platform with 300 bp paired-end kits
Data format	Raw HTS FASTQ formats
Description of data collection	Bulk soil samples were collected from each experimental pots producing Irish potatoes, irrigated with quicklime-treated AMD water (A1Q and A2Q), quicklime and fly ash- treated AMD water (AFQ), untreated AMD water (uAMD) and tap water (Control) over two cropping seasons between September 2018 and June 2019. Total DNA was purified from the replicate soil samples of each treatment using standardized methods, and DNA libraries sequenced on an Illumina MiSeq platform with 2 × 300 bp sequencing. Raw data was was processed using MOTHUR pipeline (v1.40.0) for taxonomic profiling.
Data source location	Institution: University of South Africa City/Town/Region/Country: Roodepoort, Gauteng, South Africa Latitude and longitude (and GPS coordinates) for collected samples/data: The greenhouse experiments were caried out at Ceres Greenhouse Facility, University of South Africa (UNISA), Florida Science Campus (S 26° 10′ 30″ S, 27° 55′ 22.8″ E).
Data accessibility	Repository name: NCBI Sequence Read Archive (SRA) database Data identification number: BioProject ID PRJNA974836 and Biosamples SRS17773872 to SRS17773904 (https://www.ncbi.nlm.nih.gov/bioproject/974836)
Related research article	N.A

## 1 Value of the Data

• The HTS dataset provides valuable insights into the impact of treated acid mine drainage (AMD) water on soil health and agricultural productivity in irrigated agriculture, especially considering the expected increase in AMD water usage due to global climate change.
• The metagenome datasets demonstrate the presence of diverse microbial communities and their relative abundances, including some that are unique to the samples of different AMD water irrigation treatments, thus confirming relevance and the applicability of the data in capturing the distinct microbial signatures associated with various irrigation strategies.
• By sharing the raw HTS metagenome datasets through the publicly accessible sequence repository (NCBI), researchers can use the data to identify the key factors influencing perturbations in the soil microbiome within agricultural ecosystems irrigated with treated AMD water.
• The raw HTS metagenome datasets are valuable resources for the broader scientific community, serving as a baseline for monitoring the stability of agricultural soils exposed to high heavy metal and sulfate. Researchers can utilize this data for their own research objectives.

## 2 Objective

The primary goal of the HTS sets was to delineate the complex interaction between the soil microbiome and agricultural practices, specifically focusing on the impacts of quicklime- and fly ash-treated AMD water irrigation on potato plots. The specific objectives were to comprehend the mechanisms optimizing bacterial richness and to explore the interrelationship between bacterial function and the soil environment in both treated and untreated AMD water-irrigated agriculture. The assessed quality and relevance of these HTS datasets, provide a substantial amount of information that can be harnessed for future research endeavors, enabling a deeper understanding of the soil microbiome in agricultural systems and shedding light on the potential effects of both treated and untreated AMD water irrigation treatments.

## 3 Data Description

**Fig. 1:**
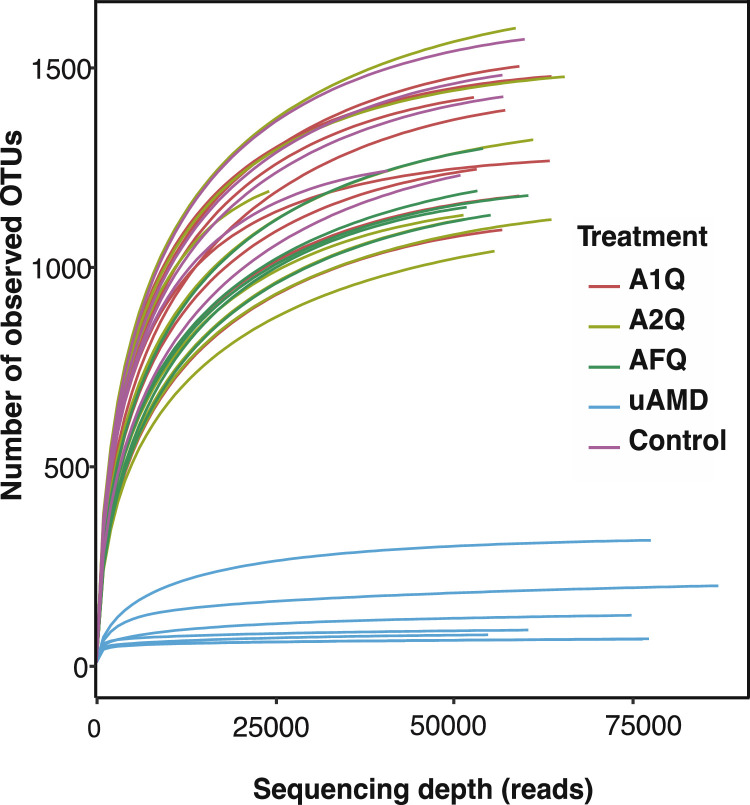
Rarefaction curves of all HTS datasets showing that the sequencing depth accurately reflected the whole bacterial communities of the soil samples. uAMD = untreated AMD water; A1Q = 1% quicklime-treated AMD water; A2Q = 2% quicklime-treated AMD water; AFQ = 1% quicklime and 25% fly ash-treated AMD water; and Control = tap water.

The HTS datasets (NCBI Bioproject ID PRJNA974836) contain microbial metagenomic data derived from soils (specifically the top 0-20 cm layer) in experimental pots dedicated to Irish potato production under greenhouse conditions. These datasets were generated from bulk soil samples exposed to various irrigation treatments, including 1% (w/v) quicklime-treated AMD water (A1Q; n = 8) [1], [2], [3], [4], [5], [6], [7], [8], 2% (w/v) quicklime-treated AMD water (A2Q; n = 7) [9], [10], [11], [12], [13], [14], [15], 1% (w/v) quicklime and 25% (w/v) fly ash-treated AMD water (AFQ; n = 5) [16], [17], [18], [19], [20], untreated AMD water (uAMD; n=7) [21], [22], [23], [24], [25], [26], [27], and tap water (Control; n=5) [28], [29], [30], [31], [32]. To obtain the metagenomic data, we conducted amplicon sequencing of the V1-V3 hypervariable segment of the 16S rRNA gene using an Illumina MiSeq platform. Table 1 presents the statistical details of the HTS data, showing a Good's coverage of >97.5% across all sample, indicating sufficient sampling depth to capture microbial diversity present in the bulk soils under different irrigation conditions. Rarefaction curves of the HTS datasets also asymptotically approached a plateau, indicating that the curves accurately reflected the whole bacterial communities (Fig. 1). This suggests that the sequencing depth adequately sampled the microbial populations in the soils under the different irrigation treatments.

**Table 1:** The high-throughput sequence statistics generated by MOTHUR (v1.40.0) pipeline. The designations of the samples are as follows: uAMD = untreated AMD water; A1Q = 1% quicklime-treated AMD water; A2Q = 2% quicklime-treated AMD water; AFQ = 1% quicklime and 25% fly ash-treated AMD water; and Control = tap water. The numbers after each sample designation represent the replicates for each irrigation treatment.

Sample	Quality reads	Average read length	Reads classified to species level	Species classified	Good's coverage	SRA Biosample ID
A1Q-1	52,894 (52.9%)	498.8 bp	35,231 (66.6%)	3,177	97.90%	SRS17773895
A1Q-2	57,294 (57.3%)	500.9 bp	39,869 (69.9%)	3,156	98.19%	SRS17773894
A1Q-3	59,272 (59.3%)	499.0 bp	42,622 (71.9%)	2,691	98.64%	SRS17773893
A1Q-4	63,629 (63.9%)	499.4 bp	42,992 (67.6%)	2,347	99.28%	SRS17773892
A1Q-5	53,332 (53.3%)	493.0 bp	39,123 (73.4%)	2,816	98.34%	SRS17773891
A1Q-6	57,166 (57.2%)	494.7 bp	42413 (74.2%)	2,636	98.48%	SRS17773890
A1Q-7	63,902 (63.9%)	501.4 bp	40,415 (63.2%)	3,044	98.78%	SRS17773904
A1Q-8	59,316 (59.3%)	500.9 bp	38,922 (65.6%)	3,395	98.20%	SRS17773903
A2Q-1	65,785 (65.8%)	501.5 bp	41,717 (63.4%)	2,875	98.91%	SRS17773876
A2Q-2	51,646 (51.6%)	496.6 bp	41,181 (79.7%)	2,514	98.24%	SRS17773878
A2Q-3	51,453 (51.5%)	494.5 bp	39,774 (77.3%)	2,418	98.37%	SRS17773879
A2Q-4	63,740 (64.4%)	496.6 bp	48,591 (76.2%)	2,694	98.77%	SRS17773880
A2Q-5	61,254 (61.3%)	496.6 bp	46,412 (75.8%)	3,055	98.49%	SRS17773883
A2Q-6	58,866 (58.9%)	503.3 bp	37,571 (63.8%)	3,633	97.86%	SRS17773899
A2Q-7	24,272 (54.3%)	498.9 bp	15,992 (65.9%)	2,311	96.39%	SRS17773897
AFQ-1	60,486 (60.5%)	497.7 bp	47,096 (77.9%)	2,745	98.64%	SRS17773889
AFQ-2	55,232 (55.2%)	499.6 bp	43,322 (78.4%)	2,489	98.43%	SRS17773886
AFQ-3	51,913 (51.9%)	501.6 bp	42,144 (81.2%)	2,543	98.41%	SRS17773885
AFQ-4	54,253 (54.3%)	497.7 bp	41,350 (76.2%)	3,081	98.06%	SRS17773884
AFQ-5	53,417 (53.4%)	496.0 bp	41,691 (78.8%)	3,107	97.88%	SRS17773882
uAMD-1	87,002 (87.0%)	529.8 bp	86,116 (99.0%	269	99.91%	SRS17773874
uAMD-2	77,591 (77.6%)	495.7 bp	59,018 (76.1%)	353	99.90%	SRS17773877
uAMD-3	60,435 (72.2%)	512.7 bp	58,472 (96.8%)	121	99.93%	SRS17773875
uAMD-4	76,384 (76.4%)	517.5 bp	74,822 (98.0%)	108	99.95%	SRS17773873
uAMD-5	74,883 (74.9%)	512.5 bp	70,441 (94.1%)	195	99.96%	SRS17773872
uAMD-6	54,805 (68.1%)	514.4 bp	51,964 (94.8%)	130	99.94%	SRS17773888
uAMD-7	77,295 (77.3%)	515.7 bp	74,369 (96.2%)	125	99.96%	SRS17773881
Control-1	56,921 (59.0%)	500.8 bp	38,146 (67.0%)	3,741	98.12%	SRS17773896
Control-2	40,814 (61.4%)	501.4 bp	27,776 (68.1%)	2,633	98.12%	SRS17773898
Control-3	60,044 (60.0%)	501.6 bp	37,531 (62.5%)	3,317	98.34%	SRS17773900
Control-4	57,056 (57.1%)	500.1 bp	38,817 (68.0%)	3,357	98.18%	SRS17773902
Control-5	51,005 (51.0%)	495.0 bp	39,645 (77.7%)	3,135	97.58%	SRS17773887

**Fig. 2:**
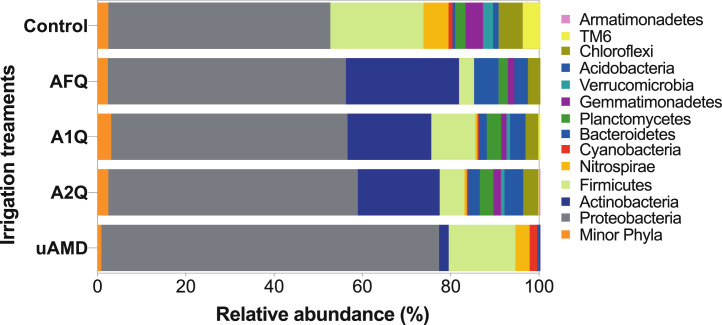
Relative abundance distribution of major taxa (>1% abundance) at the phylum level of the microbial community from bulk soils irrigated with (un)treated AMD water. Sample designation and replication was as follows: uAMD = untreated AMD water (n=7); A1Q = 1% quicklime-treated AMD water (n=8); A2Q = 2% quicklime-treated AMD water (n=7); AFQ = 1% quicklime and 25% fly ash-treated AMD water (n=5); and Control = tap water (n=5). The taxonomic identities were assigned based on the amplicon sequencing of the V1-V3 hypervariable segment of the 16S rRNA gene through the SILVA v132 database [33].

The metagenomic data at the phylum level revealed notable differences in abundance among different soil samples. *Proteobacteria* (76.5%), *Firmicutes* (15.1%), *Nitrospirae* (3.21%), *Cyanobacteria* (1.68%), and *Bacteroidetes* (0.44%) were found to be more abundant in AMD soil samples compared to the other treatments (Fig. 2; Table 2). However, the other soil samples exhibited a more diverse microbial community compared to the uAMD samples. Specifically, *Acidobacteria, Planctomycetes, Chloroflexi, Gemmatimonadetes, Verrucomicrobia*, TM6, and *Armatimonadetes* were not detected in the AMD samples. In contrast, A1Q, A2Q, AFQ, and Control samples showed higher abundances of *Actinobacteria* (18.6-25.7%), *Acidobacteria* (3.08-5.46%), *Bacteroidetes* (1.81-5.59%), *Chloroflexi* (2.49-3.71%), and *Gemmatimonadetes* (1.22-2.23%), alongside *Proteobacteria* (50.3-56.5%) and *Firmicutes* (3.33-9.95%) as the major taxa. The remaining phyla exhibited values of 1% or lower in abundance. The distribution of the major phyla and their relative abundances, as depicted in Figure 2 and summarized in Table 2, further strengthens the reliability and suitability of the HTS datasets generated. The dataset highlights the distinct microbial composition in the AMD soil samples and the differences observed in the microbial community structure among the various irrigation treatments. Overall, the dataset's ability to capture and differentiate these microbial patterns underscores its significance for studying the impacts of (un)treated AMD water irrigation strategies on soil microbiomes in agricultural systems.

**Table 2:** Relative abundance of the representative high-throughput sequences from bulk soils irrigated with (un)treated AMD water. Percentages were calculated across the sample groups: uAMD = untreated AMD water; A1Q = 1% quicklime-treated AMD water; A2Q = 2% quicklime-treated AMD water; AFQ = quicklime and fly ash-treated AMD water; and Control = tap water.

Phylum	uAMD	A2Q	A1Q	AFQ	Control
Proteobacteria	76.47%	56.49%	53.53%	53.87%	50.27%
Actinobacteria	2.18%	18.56%	19.01%	25.66%	21.09%
Firmicutes	15.13%	5.59%	9.95%	3.33%	5.73%
Acidobacteria	0.00%	4.23%	3.49%	3.08%	5.46%
Bacteroidetes	0.44%	2.59%	1.81%	5.59%	2.23%
Planctomycetes	0.00%	3.05%	3.31%	2.10%	4.06%
Chloroflexi	0.00%	3.36%	2.92%	2.49%	3.71%
Gemmatimonadetes	0.00%	1.76%	1.22%	1.49%	2.23%
Nitrospirae	3.21%	0.52%	0.50%	0.00%	0.87%
Cyanobacteria	1.68%	0.36%	0.26%	0.00%	0.66%
Verrucomicrobia	0.00%	0.82%	0.77%	0.00%	1.25%
TM6	0.00%	0.15%	0.15%	0.00%	0.00%
Armatimonadetes	0.00%	0.06%	0.00%	0.00%	0.00%
Minor phyla	0.90%	2.46%	3.07%	2.38%	2.44%

## 4 Experimental Design, Materials and Methods

### 4.1 Experimental Design and Sampling

AMD water samples were collected from mine tailing dam of Sibanye Gold Mine located in Randfontein, Gauteng Province, South Africa as previously described [34]. AMD water was subjected to three different treatments with quicklime and fly ash before use as irrigation water in the experiments: 10g quicklime + 990 mL AMD water (A1Q); 20g quicklime + 980 mL AMD water (A2Q); and 10g quicklime + 250 g fly ash + 740 mL AMD water (AFQ). The AMD water was treated with quicklime and fly ash based on the protocol by Othman et al. [35].

The greenhouse experiment was caried out at Ceres Greenhouse Facility, University of South Africa (UNISA), Florida Science Campus, Roodepoort, Johannesburg, Gauteng Province (S 26° 10′ 30″ S, 27° 55′ 22.8″ E). The experiment was conducted for two cropping seasons, from September to December 2018 (Season 1) to March to June 2019 (Season 2). The layout of the experiment was a completely randomized block design with at least five replications for each of the irrigation water (Control, AMD, A1Q, A2Q and AFQ) tested for each potato cultivar. Potatoes were sown in 5 L pots filled with 3:1:1 Culterra topsoil + vermiculite + river sand, the typical soil composition in the Gauteng region, South Africa. The typical physicochemical parameters of the soil substrate at the start of the experiment were as follows: pH 7.26, bulk density (BD) 1.23 g cm−3, 15.63 g kg−1 soil organic carbon (SOC), 0.81 g kg−1 total nitrogen (TN), 33.2 mg kg−1 available phosphorus (AP), and 163.3 mg kg−1 available potassium (AK). Potato tubers were planted in each experimental pot at a depth of 7.5 cm and irrigated with 500 mL water after every 2 days until harvesting. The mean temperatures, relative humidity, and light intensity during the study period were 25°C, 50%, and 1100 µmol s^−1^ m^−2^.

### 4.2 High-Throughput Sequencing and Taxonomic Profiling

Metagenomic DNA was extracted from triplicates soil samples for each treatment, each weighing 0.5 g, using the PowerSoil ® DNA isolation kit (MoBio Laboratory, CA, USA) according to the manufacturer's instructions. A PCR amplification to cover the bacterial 16S rRNA V1-V3 hypervariable region was performed using 27F (5’-AATGATACGGCGACCACCGAGATCTACAC**TATGGCGAGTGA**AGAGTTTGATCMTGGCTCAG-3’) and 519R (5’-CAAGCAGAAGACGGCATACGAGAT**AGTCAGTCAGGG** GWATTACCGCGGCKGCTG-3’) primer pairs, fused with MiSeq adapters (underlined) and heterogeneity spacers (bolded) compatible with Illumina indexes for multiplex sequencing, as described by Ogola et al. [36]. The generated libraries were sequenced using the Illumina MiSeq 2 × 300-bp paired-end platform at the University of South Africa – Florida Science Campus. The raw sequences of the HTS datasets have been deposited to the National Center for Biotechnology Information (NCBI) Sequence Read Archive (SRA) under Bioproject ID PRJNA974836 (https://www.ncbi.nlm.nih.gov/bioproject/974836).

The raw fastq data files obtained from MiSeq sequencing platform were initially processed to remove PCR artefacts, Illumina tags, and low-quality reads. This trimming process was performed using the *ngsShoRT* trimmer algorithm [37]. The resulting trimmed reads were then merged and further processed using the MOTHUR version 1.40.0 pipeline [38]. Within the MOTHUR pipeline, the sequence reads underwent quality filtering, ensuring that only high-quality reads were retained. Chimeric sequences and potential eukaryotic contaminants were identified and removed using the UCHIME algorithm [39]. To classify the remaining sequences, the Naïve Bayesian classifier algorithm [40] was employed, utilizing the SILVA database version 132 [33] as the reference database. Operational taxonomic units (OTUs) were determined by grouping the aligned sequences based on a pairwise distance matrix using the Euclidean distance measure [38]. The clustering was performed at a sequence similarity threshold of 97% at various taxonomic levels, including phylum, class, order, family, and genus.

## CRediT authorship contribution statement

**Henry Joseph Oduor Ogola:** Software, Visualization, Investigation, Writing – review & editing. **Rabelani Munyai:** Conceptualization, Methodology, Writing – review & editing. **Ramganesh Selvarajan:** Data curation, Supervision, Writing – review & editing.

## Data Availability

Microbial diversity of (un)treated acid mine water irrigated soils under potato production (Bioproject ID PRJNA974836) (Original data) (NCBI).
